# Assessing Fishing and Marine Biodiversity Changes Using Fishers' Perceptions: The Spanish Mediterranean and Gulf of Cadiz Case Study

**DOI:** 10.1371/journal.pone.0085670

**Published:** 2014-01-22

**Authors:** Marta Coll, Marta Carreras, Cristina Ciércoles, Maria-José Cornax, Giulia Gorelli, Elvira Morote, Raquel Sáez

**Affiliations:** 1 Institut de Ciències del Mar (ICM-CSIC). Barcelona, Spain; 2 UMR EME 212 Centre de Recherche Halieutique Méditerranéenne et Tropicale. IRD - IFREMER & Université Montpellier II. Sète Cedex, France; 3 OCEANA, Madrid, Spain; 4 Investigación, Planificación y Desarrollo S.A., Madrid, Spain; 5 Universidad de Almería (UAL). Almeria, Spain; Aristotle University of Thessaloniki, Greece

## Abstract

**Background:**

The expansion of fishing activities has intensively transformed marine ecosystems worldwide. However, available time series do not frequently cover historical periods.

**Methodology:**

Fishers' perceptions were used to complement data and characterise changes in fishing activity and exploited ecosystems in the Spanish Mediterranean Sea and Gulf of Cadiz. Fishers' interviews were conducted in 27 fishing harbours of the area, and included 64 fishers from ages between 20 to >70 years old to capture the experiences and memories of various generations. Results are discussed in comparison with available independent information using stock assessments and international convention lists.

**Principal Findings:**

According to fishers, fishing activity substantially evolved in the area with time, expanding towards deeper grounds and towards areas more distant from the coast. The maximum amount of catch ever caught and the weight of the largest species ever captured inversely declined with time. Fishers (70%) cited specific fishing grounds where depletion occurred. They documented ecological changes of marine biodiversity during the last half of the century: 94% reported the decline of commercially important fish and invertebrates and 61% listed species that could have been extirpated, with frequent mentions to cartilaginous fish. Declines and extirpations were in line with available quantitative evaluations from stock assessments and international conventions, and were likely linked to fishing impacts. Conversely, half of interviewed fishers claimed that several species had proliferated, such as cephalopods, jellyfish, and small-sized fish. These changes were likely related to trophic cascades due to fishing and due to climate change effects. The species composition of depletions, local extinctions and proliferations showed differences by region suggesting that regional dynamics are important when analysing biodiversity changes.

**Conclusions/Significance:**

Using fishers' perceptions, fishing and ecological changes in the study area were documented. The recovery of local ecological knowledge provides valuable information complementing quantitative monitoring and evaluation surveys.

## Introduction

From coastal areas to the open seas, human activities impact marine ecosystems [1], [2]. In the past, human impacts on marine ecosystems were mainly due to exploitation of marine resources and fishing. These activities diversified with time and currently include other impacts such as habitat degradation, pollution and eutrophication, climate change, and the invasion of non-indigenous species [3]–[6]. These impacts have an effect on the biodiversity and abundance of marine resources [7]–[9], and on marine ecosystem structure and functioning [10], [11].

The Mediterranean Sea is one of the most impacted marine ecosystems worldwide [5], [12]–[14]. Exploitation and habitat loss are the main human activities shaping its biodiversity changes [12]. Fishing impacts are notable and several commercial species are highly exploited or overexploited [15], [16]. Non-commercial species have also shown important declines [16], [17].

Additionally, the impact of widely distributed factors such as climate change or the invasion of non-indigenous species is steadily increasing [18]–[20]. Human impacts are distributed in a heterogenic way along the Mediterranean Sea and many areas are subjected to multiple impacts simultaneously [13]. Some of the most impacted areas are located in the western Mediterranean Sea region [13].

The information on past ecosystems and changes in marine biodiversity is quickly lost because of perception change (this is known as “shifting baselines” syndrome) [21]–[23]. This is even more evident in ecosystems where changes have occurred centuries ago, such as the Mediterranean Sea [24]. Therefore, several studies have aimed at recovering knowledge from past Mediterranean ecosystems to reconstruct ecosystem baselines [14], [25]. The loss of information from the past is also due to the availability of long time series of data is frequently scarce. Data only started to be regularly available from the early 1990s [e.g. 26], after important changes in marine ecosystems had already occurred [14], [17], [25].

To overcome some of the limitations in data availability and to recover historical information, scientists have documented changes in marine ecosystems using fishers' perceptions (also called Local Ecological Knowledge, LEK, or Traditional Ecological Knowledge, TEK) [27]–[31]. In the Mediterranean Sea, applications to recover this knowledge have yield interesting results, such as information on depletion of large predators [32] and on changes in marine fish diversity due to meridionalization [33].

In this study we used fishers' perceptions to complement available information on (i) fishing changes with time, and (ii) ecological changes of marine ecosystems in the most western Mediterranean area: the Spanish Mediterranean Sea. We extended our study area to include the Gulf of Cadiz (Atlantic adjacent waters to the Mediterranean Sea) due to the fact that both fisheries and ecosystems share similar features [34]. In addition, and to contrast the information obtained with traditional knowledge, we discussed our results in comparison with available independent quantitative information using stock assessments and international convention lists. This comparison allowed us to evaluate the utility of this traditional knowledge with the aim to reconcile it with data formally collected by fisheries scientists.

## Materials and Methods

### Ethics statement

The study followed all the guidelines of the ethics committee of CSIC and available legislation (*Comité de Ética CSIC*, http://www.csic.es/web/guest/normativa). The interviews included a first page with the objectives of the research and contact information, which was read out loud to the participants before starting the interview. All interviews took place within the country and participants provided verbal consent to participate in the study by agreeing to participate in the interview. Participants could decide either to have their names written down in the first page of the interview or to leave it anonymous.

### Study area

**Figure 1:**
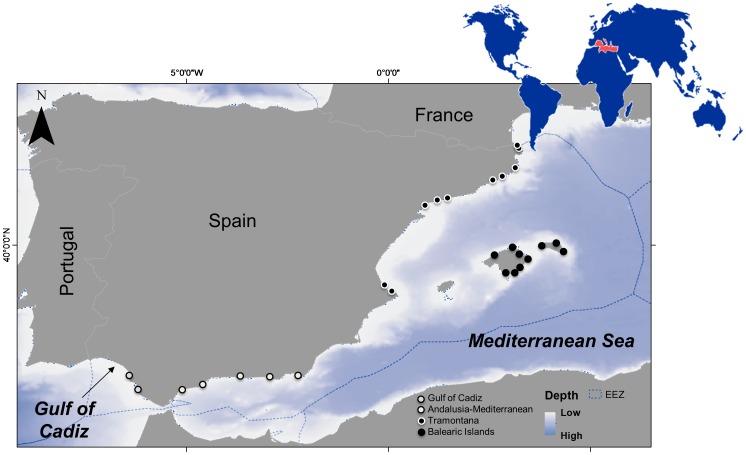
Study area in the Spanish Mediterranean Sea and Gulf of Cadiz. Harbours where interviews were conducted are indicated.

Our study area includes the Spanish Mediterranean (Figure 1), and it is extended to the Gulf of Cadiz, in the immediate Atlantic waters outside the Mediterranean Sea. This region is located in the Balearic FAO area 37.1.1 and the South-East Atlantic area 27.IXa. The area is limited by the Gulf of Lions in the north, Moroccan waters in the south and the Sardinia FAO area 37.1.3, including the Tyrrhenian Sea and adjacent waters to the east.

The continental shelf of the Spanish Mediterranean and Gulf of Cadiz regions is generally narrow and the 50 m depth is usually found approximately three nautical miles from shore (Figure 1). Fishing activities are mainly coastal or littoral, and they last approximately 12 hours a day [34]. The main fishing fleets operating in the area are bottom trawling (∼24% of the fleet), purse seine (∼9%), bottom and surface long liners (∼3%), and a diverse artisanal fleet, including gillnets (∼64%) [34]. The area contains 95 fishing harbours, more than 90 first-source fishing markets (where catches that are unloaded in the harbour are commercialized), and several fishers brotherhoods [fishers labour organizations unique to Spain that date back to early Medieval times, 35].

In 2008–2009, the total number of fishing vessels in the study region was 4 140, with total declared power of 453 593 Kw and a mean total length of 12 meters [36]. In late 2000s, more than 13 000 people directly worked as fishers in the area [36]. The area is inhabited by approximately 24 million people.

Major species in the official landings of the area include sardine (*Sardina pilchardus*), anchovy (*Engraulis encrasicolus*), and other small and medium-sized pelagic fish such as round sardinelle (*Sardinella aurita*), horse mackerel (*Trachurus* spp.) and mackerel (*Scomber* spp.), and demersal species, such as common hake (*Merluccius merluccius*), red mullets (*Mullus* spp.), anglerfish (*Lophius* spp.) and blue whiting (*Micromesistius poutassou*). Invertebrate catches are also economically important such as, for example, that of red shrimp (*Aristeus antennatus*), European spiny lobster (*Palinurus elephas*), rose shrimp (*Parapenaeus longirostris*), Norway lobster (*Nephrops norvegicus*) and various cephalopod species [37], [38]. The Mediterranean Sea also yields important catches of tunas and associated species, especially of Atlantic Bluefin tuna (*Thunnus thynnus*) and swordfish (*Xiphias gladius*) [34].

### Fishers' interviews

This study was conducted within a larger project aiming at collecting available information to better estimate total fisheries removals from the area. Individual interviews with selected fishers were developed and executed in order to get information on different aspects of fishing activities and mechanisms to commercialize landed catches, and including recreational and artisanal fishing, discarding and illegal fishing, and black market practices [34], [39]. In addition, we retrieved information on fishers' perceptions regarding (i) fishing changes with time, and (ii) ecological changes of marine ecosystems.

Fishers were interviewed from 27 harbors distributed in four different regions (Figure 1): the Gulf of Cadiz (in the Atlantic waters adjacent to the Mediterranean Sea) (with n = 14 fishers interviewed), the Mediterranean Andalusia region (n = 12), Tramontana region (in the northern Spanish Mediterranean area) (n = 20) and the Balearic Islands (n = 18). Fishers were all professional skippers and crew of the fishing fleet. Interviewees were chosen due to they were deemed reliable to provide objective information since previous relationships had been directly or indirectly established with them. This was deemed important because the aim was to minimize uninformative and low quality answers.

The interviews included a series of open or fixed questions, some of them using multiple-choice and with quantitative or qualitative (yes-no) answers. First a series of questions targeted to retrieve specific information from the fisher (such as age, year of starting fishing, fishing gear used, species targeted) were posed. Secondly, fishers were asked a series of questions regarding fishing activity and the following questions were posed: (1–2) What was the depth/distance from the coast you used to fish when you started fishing, and what is the depth/distance you fish at present; (3) What was your highest catch in a day of fishing and when was it (year)? (4) What was the weight of the largest species that you ever caught, what was it (species), and when was it (year)? (5) Do you know of any locations where you fish that are depleted? and (6–7) What was the weight of the largest common hake ever caught and when was it (year)? Finally, and regarding ecosystem changes, fishers were asked: (8–9) Do you know of any species in the area that had declined/disappeared during the time you have been fishing? And (10) Do you know of any species in the area that had increased during the time you have been fishing? Faunistic guides or species landed in the harbor at the same time of the interviews were used to confirm the identification of species mentioned by fishers during the interviews.

To contrast the information obtained with traditional knowledge, we compared results with available independent quantitative information using stock assessments and international convention lists from: (1) The International Union for Conservation of Nature and Natural Resources (IUCN) [16], (2) The Bern Convention (or Convention on the Conservation of European Wildlife and Natural Habitats, 1979) [40], (3) The Barcelona Convention (or Convention for the Protection of the Marine Environment and the Coastal Region of the Mediterranean, 1976, amended in 1995, and the Protocol Concerning specially Protected Areas and Biological Diversity in the Mediterranean, SAP-Bio, 1995) [41], (4) the Bonn Convention (or Convention on the Conservation of Migratory Species of Wild Animals 1983) [42], (5) The CITES list (or Convention on International Trade in Endangered Species of Wild Fauna and Flora, 1975) [43], and (6) Assessments from the General Fisheries Commission for the Mediterranean Sea - Scientific Advisory Committee [44].

### Statistical analysis

We used three non-parametric tests to analyze our results with the aim to avoid problems with the asymmetrical distribution of the data, to maximize the robustness of our results and to minimize the probability to commit type I errors. The non-parametric Mann–Whitney U test was used to compare the data obtained regarding fishing at different depths and distances. This analysis was developed with all data from all fishers regardless their age, and by separating the age in two groups: younger than 40 years or older. The non-parametric Spearman rank's correlation was used to detect correlation between quantitative data, such as weight of catch or caught species and year. Information about the year was used as year reporting the specific quantity (reported year) and year that fishers started fishing. This was due to the fact that in some cases fishers did not remember the reported year, but they did remember when they started fishing.

To investigate differences in the species composition list of depletions, local extinctions and proliferations between regions, the non-parametric multivariate permutational analysis of variance (PERMANOVA, in PRIMER with PERMANOVA+ v. 6, PRIMER-E Ltd., Plymouth, UK) on the Euclidean distance matrix was employed. PERMANOVA calculates a pseudo-F statistic that is directly analogous to the traditional F-statistic for multifactorial univariate ANOVA models, but uses permutation procedures (here 9999 permutations) to obtain p-values for each term in the model [45]. We performed a 1-way analysis with the region factor and the list of depletions, local extinctions and proliferations, separately. We considered that each interview from the same location was an independent sample and the location factor was divided in four levels: the Andalusia-GoC (Gulf of Cadiz in the Atlantic region), the Andalusia-Mediterranean (southern Mediterranean region), the Tramontana area (northern Spanish coast) and the Balearic Islands (Figure 1). The Similarity Percentages Procedure (SIMPER) [46] was used when results from PERMANOVA were significant to identify the species that were most important in each region regarding depletions, local extinctions and proliferations.

## Results

### Interviewed fishers

**Figure 2:**
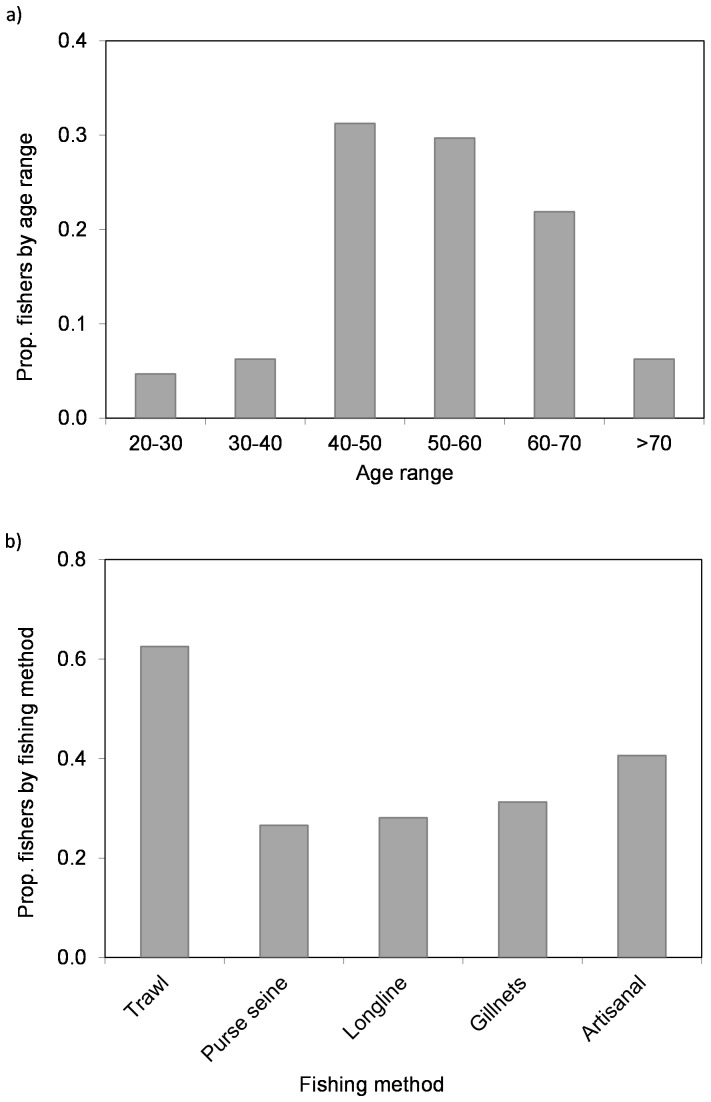
Proportion of a) fishers interviewed by age range (n = 64), and b) fishing methods used by fishers. In Figure 2b fishers can be represented more than once due to results integrate their knowledge through their entire working life, thus the total proportions do not sum to 1.

During 2011 and 2012, a total of 64 fishers were interviewed. Fishers' ages were from 20 to >70 years old (Figure 2a). Fishers started fishing from 1939 to 2005, thus covering more than 70 years of observations as a result of the experience of the interviewees (Figure 2a).

Most of interviewed fishers had been involved in different fishing segments during their lives and most of them were fishing in the trawling fleet, but had also participated in other fishing activities (Figure 2b). Fishers were targeting a variety of highly commercial species such as red mullets (52%), common hake (45%), anglerfish (42%), red shrimp (42%), European anchovy (31%) and European sardine (30%). Overall, 37 fish and invertebrate species were listed as targeted (Table S1). This highlights the multispecificity of the fishing activity in the area (Figure 2b).

### Fishing activity

**Figure 3:**
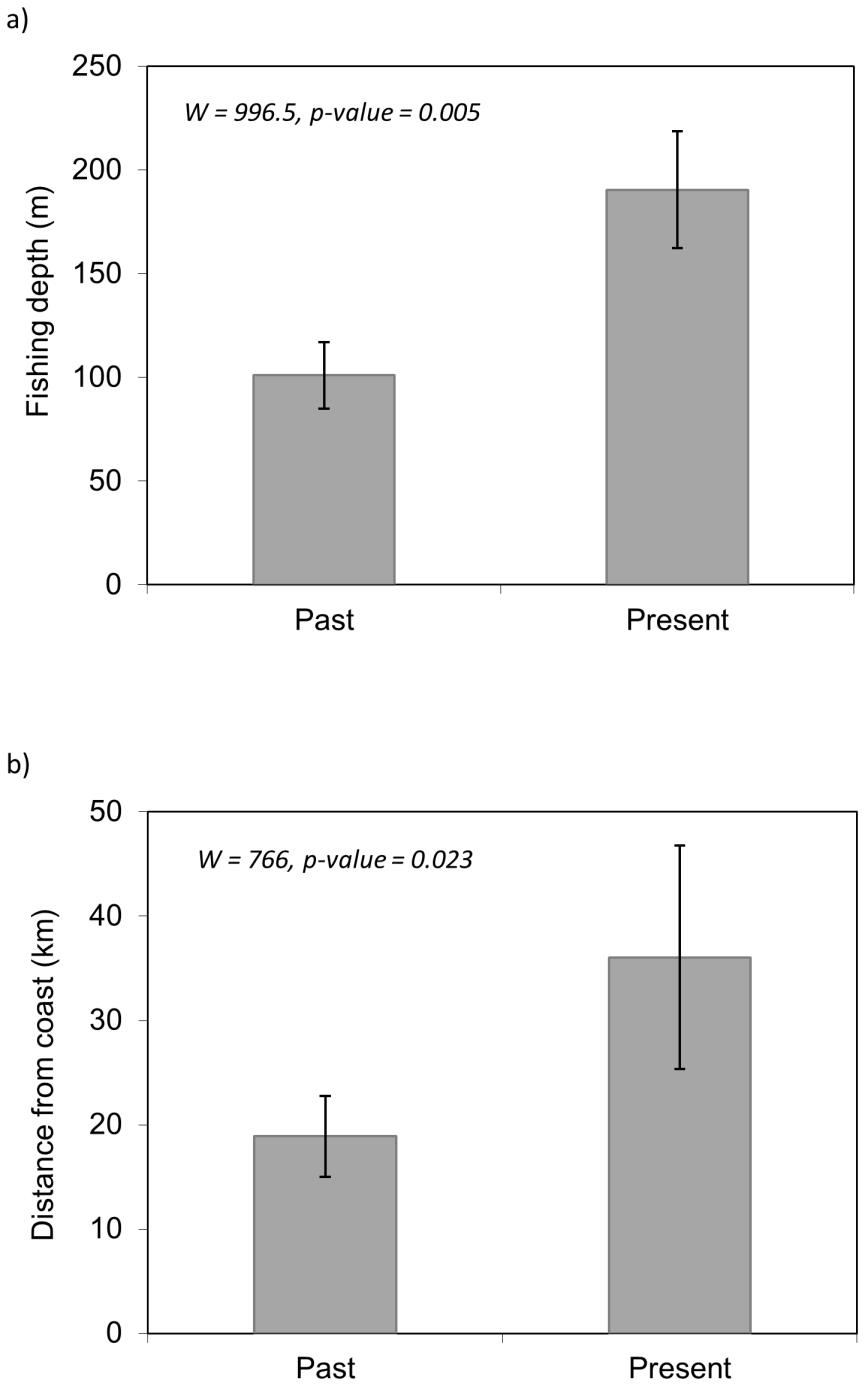
Mean and standard deviation of a) depth (m) and b) distance from the coast (km) between past and present fishing activities.

- **Fishing depth and distance**: Results showed a clear and significant difference between the depth and distance that fishers used to fish when they started fishing, compared to where they fish at present (Figures 3a and b). Thus, both the present depth and distance from the coast were significantly larger now than when fishers started fishing (p-value  = 0.005 and p-value  = 0.023, respectively for depth and distance). The range of values at present was wider than in the past for both depth and distance.

**Figure 4:**
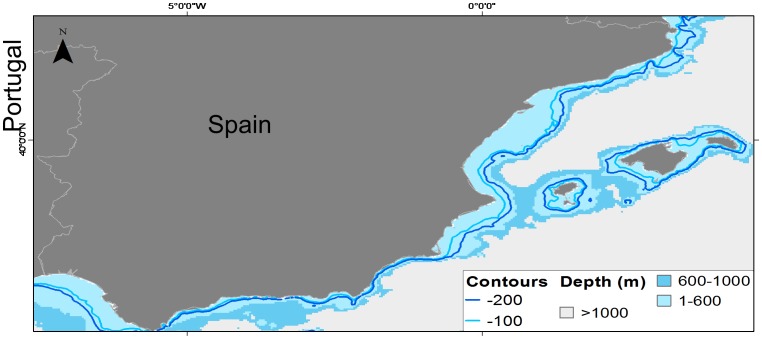
Spatial representation of mean depth and range values between past (mean  = 100, range  = 1–600 m) and present (mean  = 200, range  = 1–1000 m) fishing activities.

The spatial representation of these results illustrates how the fishing activity has potentially expanded from coastal and shelf areas to deeper areas that are more distant from the coast (Figure 4). While in the past the mean value of fishing was 100 m depth, at present fishers are working at mean depths of 200 m. The range values also expanded substantially, from 1–600 m in the past to 2–1000 m at present (minimum and maximum values reported, respectively). Similarly, the mean distance reported by fishers in the past was 19 km, while this value increased to 36 km in the present. The range of distances also shows an expansion of the fishery, from 1–111 km to 1–370 km in the present.

**Figure 5:**
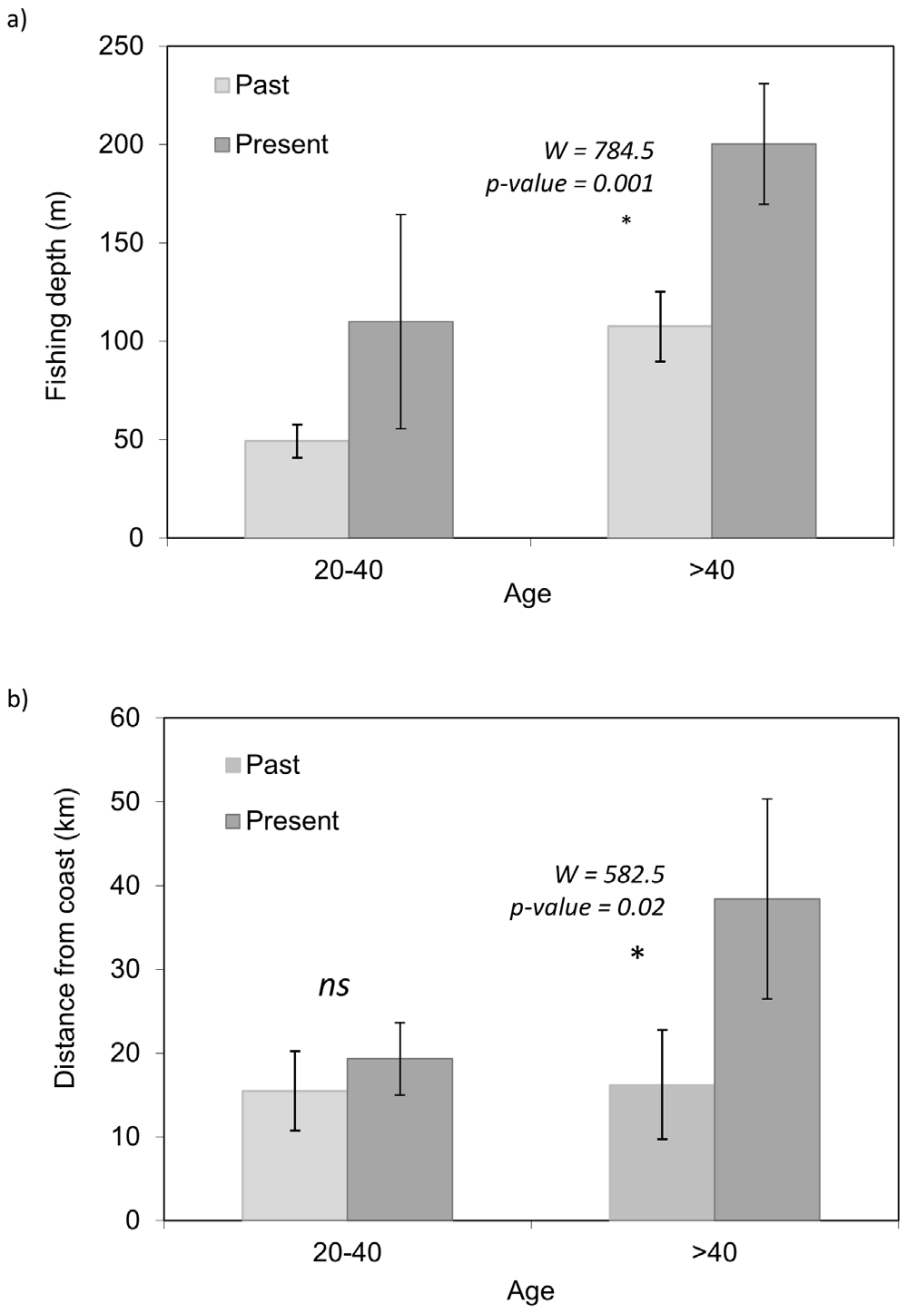
Mean and standard deviation of a) depth (m) and d) distance from the coast (km) between past and present fishing activities by age group (fishers younger or older than 40 years old).

Interestingly, when separating results by the age of fishers in two groups (younger or older than 40 years), significant difference between the depth and distance that fishers used to fish in the past compared to where they fish at present was significant only for fishers with older ages (Figures 5a and b).

**Figure 6:**
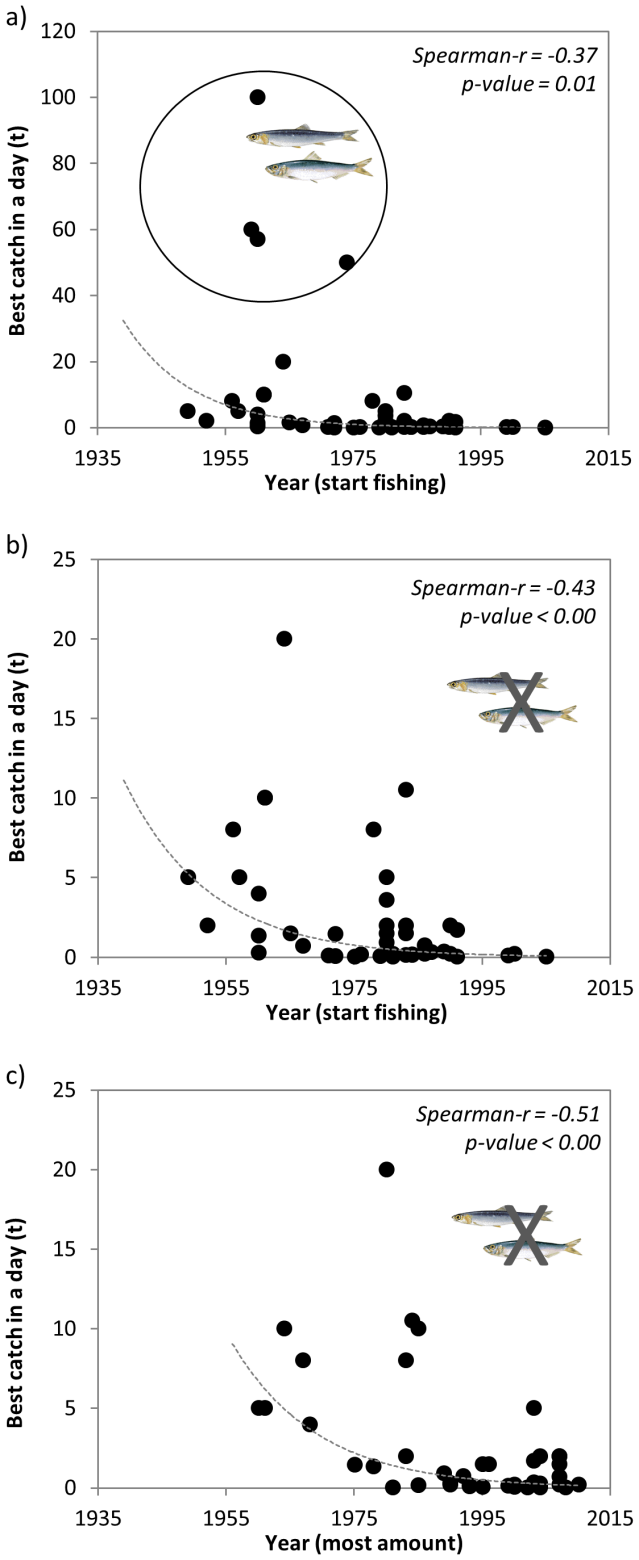
Assessment of the a) best catch in a day of fishing (t) and year of starting fishing (n = 47), b) best catch in a day of fishing (t) and year of starting fishing, excluding small pelagic fish and squids (n = 43), and c) best catch in a day of fishing (t) (n = 43) and year of the best catch. (Species drawings: www.gencat.cat).

- **Highest catch in a day of fishing and largest species ever caught:** The fishers were asked to estimate the highest catch in metric tonnage (t) in a day of fishing and when was it (year). Results showed a significant decline (Spearman r = −0.37, p-value  = 0.01) with time on the largest amount caught in a day of fishing (best catch) analysed against the year that fishers started fishing (Figure 6a). Since fishers frequently mentioned the catch of small pelagic fish and squids as the total amount of boxes being landed, these boxes had to be converted into total amount of tonnes with approximations given by fishers. When those values reported in boxes of small pelagic fish and squids were removed from the analysis the declining pattern was even clearer (Spearman r = −0.43, p-value <0.00) (Figure 6b). This may be due to the fact that small pelagic fish and squids depend more strongly than demersal organisms on the effects of environmental fluctuations. Results were similar (Spearman r = −0.51, p-value <0.00) when these data was compared against the year when the highest catch occurred (Figure 6c). Both results showed that while from the 1950s to 1980s the best catch could be as high as 10 tonnes per day, in the 1990s and 2000s it was always below 5 tonnes.

**Figure 7:**
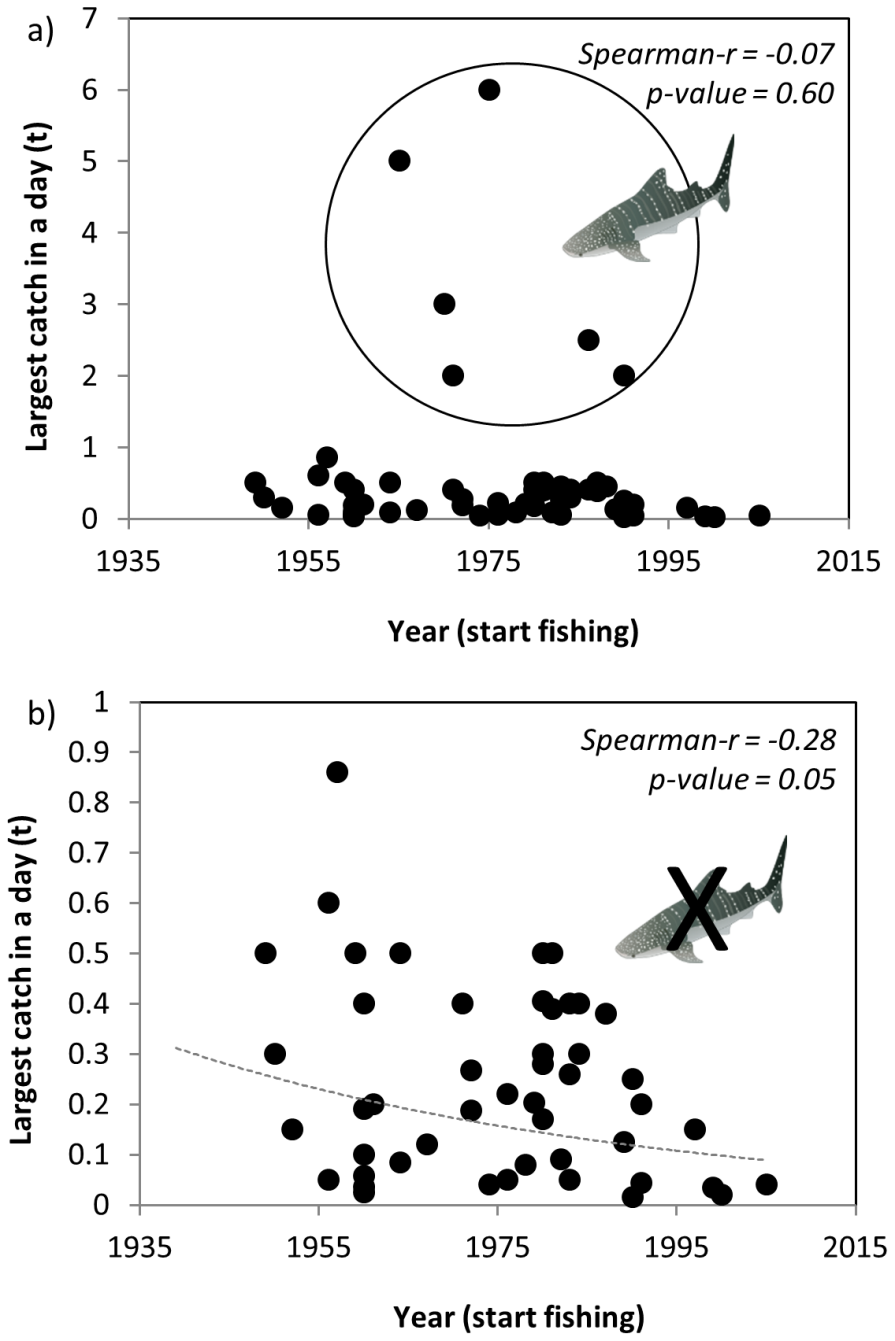
Assessment of the a) largest caught species in a day of fishing (t) and year of starting fishing (n = 58), and b) largest caught species in a day of fishing (t) and year of starting fishing, excluding whale and basking sharks (n = 56). (Species drawings: http://ian.umces.edu/symbols).

The fishers were also asked to estimate the weight (t) of the largest species ever caught, what was it (species), and when was it (year). Results when looking at the largest species ever caught against the year of start fishing showed no clear results (Figure 7a). However, when removing individuals of whale shark *Rhincodon typus* and basking shark *Cetorhinus maximus* (both large filter feeders) from the analysis, a negative trend emerged (Spearman r = −0.28, p-value  = 0.05) (Figure 7b). Filter feeders may have benefited in the past from larger amounts of prey in the ecosystem due to trophic cascades or environmental fluctuations and thus this second analysis wanted to remove this potential effect. When analyzing these results by decade to aggregate the data, results illustrate that while fishermen starting fishing from the 1940s to the 1960s had caught species of half a ton, the largest species ever caught by young fishers in the area is of less than 200 kg. This result illustrates that the biggest species ever caught seemed to be smaller in recent years.

**Figure 8:**
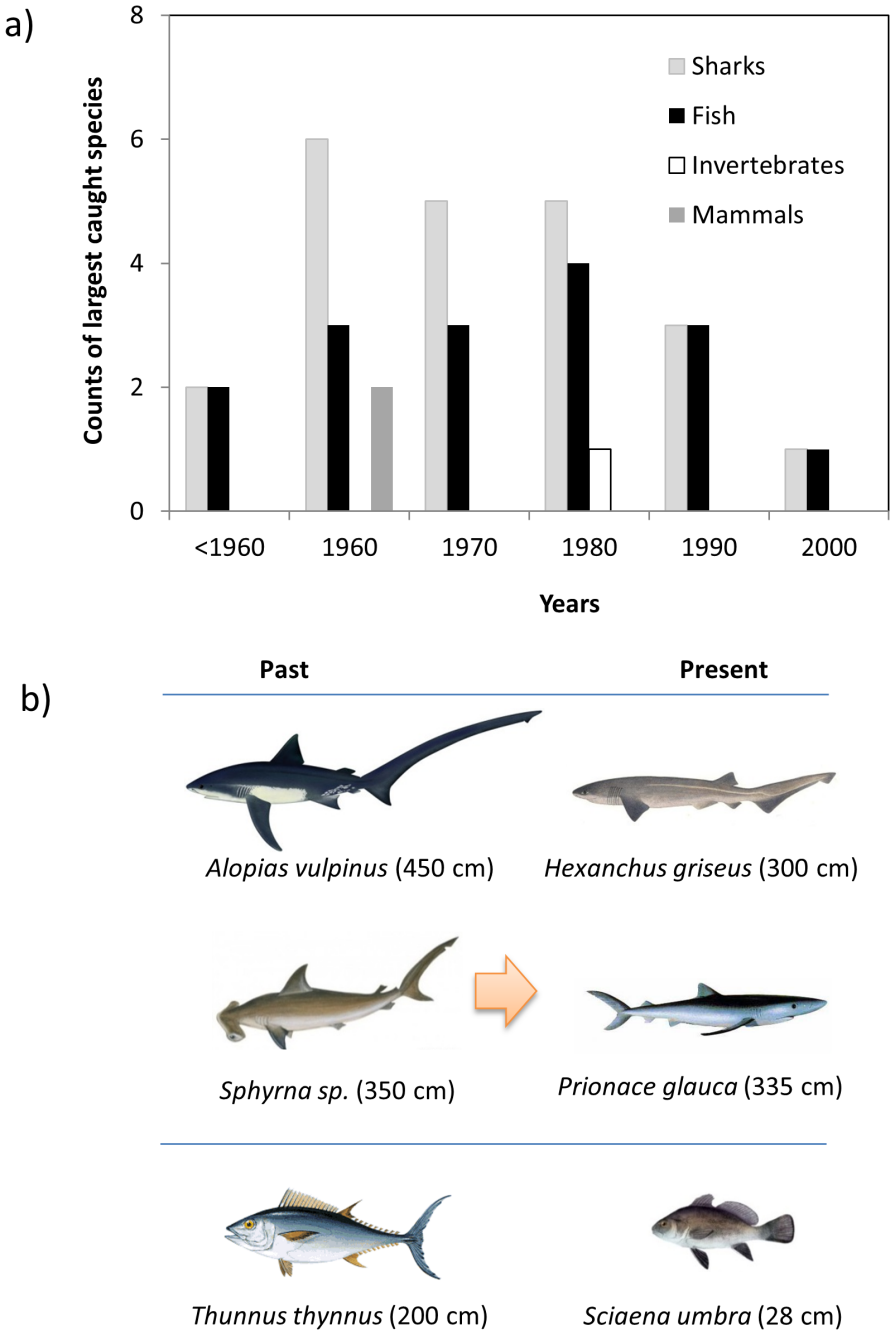
Composition of the largest caught species listed by fishers a) by decade and main species group, and b) main cartilaginous and bony fish species reported during the first and last decades and their mean length (species drawings:http://ian.umces.edu/symbols & www.gencat.cat).

The composition of the species that were reported to be the largest ever caught changed with time (Figure 8a). Shark species were the most cited until the 1980s. Fish species increased from the 1940s until the 1980s, but declined afterwards. Marine mammals were only mentioned in the 1960s and invertebrate species in the 1980s. In addition, the mean size composition of the largest fish and sharks ever caught [considering the mean sizes give in 47] mentioned by fishers also changed to smaller sizes with time, from larger sharks (such as the common thresher shark *Alopias vulpinus* and hammerhead sharks *Sphyrna* sp.) and fish (bluefin tuna) to smaller sharks (such as the bluntnose sixgill shark *Hexanchus griseus* and the blue shark *Prionace glauca*), and smaller fish (such as brown meagre *Sciaena umbra*) (Figure 8b).

**Figure 9:**
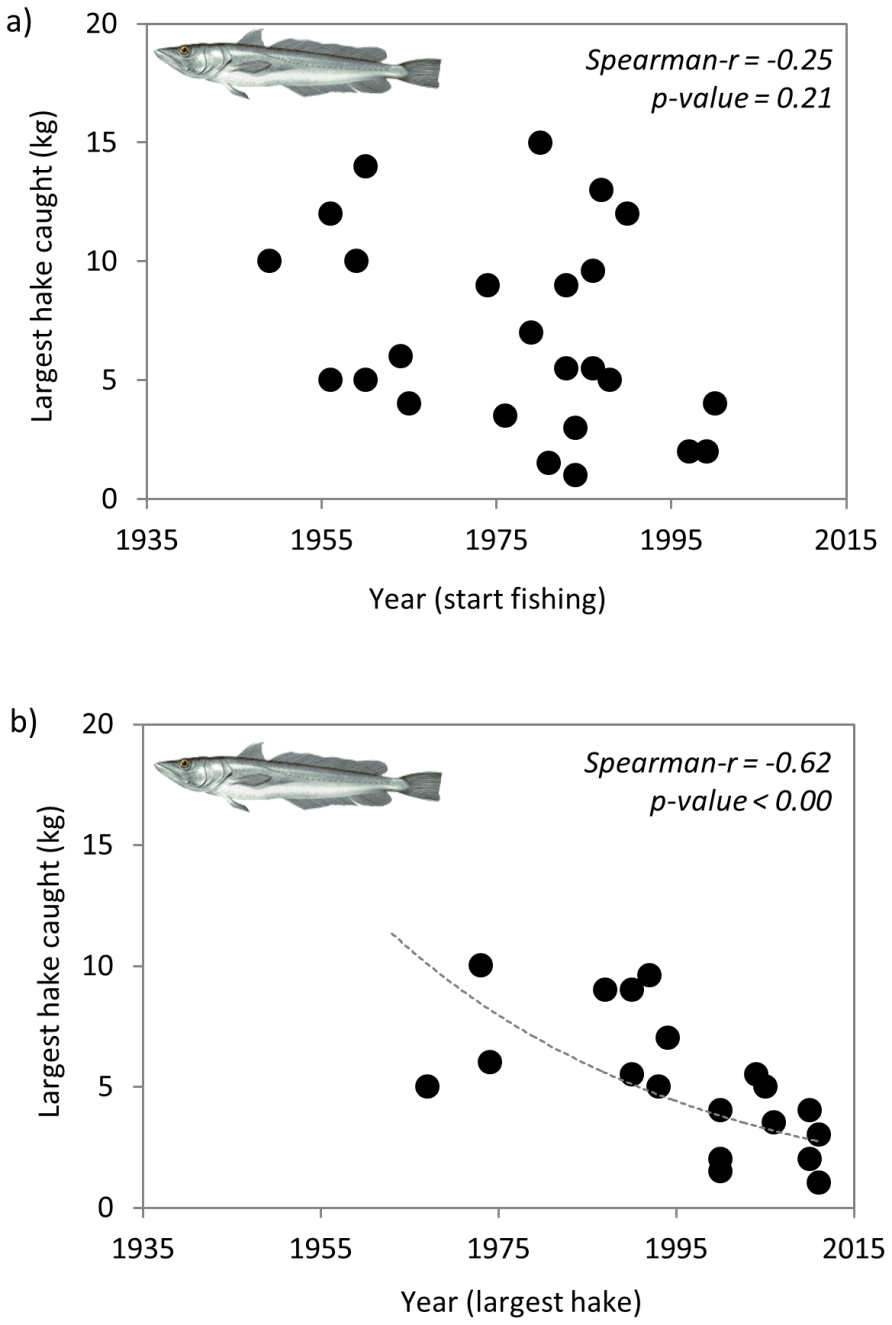
Largest common hake individuals caught (kg) by a) years of starting fishing (n = 30) and b) year of the largest caught individual (n = 25). (Species drawings: www.gencat.cat).

Common hake is highly targeted in the study areas by trawlers but also by long liners and artisanal fisheries. Results on the weight of the largest common hake ever caught (kg) plotted against the year of starting fishing showed no clear trend (Figure 9a). However, a negative relationship emerged (Spearman-r  = −0.62, p-value <0.00) when plotting the data with the year of the largest hake ever caught, despite this dataset was shorter because several fishermen did not remember the year they caught their largest hake.

- **Expansion, depletion and shifting baselines:** Overall, the majority of fishers knew several sites that had been depleted (Table 1). Of those knowing depleted sites, 34% of them highlighted that “all” sites where they fished were depleted and 5% mentioned that “many” sites had been depleted. Another 31% listed specific locations in their fishing grounds that had been depleted.

**Table 1:** Summary of answers from fishers' interviews.

Sites	Yes	No	NK-NA
Depletions	70%*	2%	28%

Depletions	94%	2%	4%
Extinctions	61%	27%	12%
Proliferations	53%	36%	11%

* “All”: 34%, “Many”: 5%, Specific sites: 31%. NK-NA: not known, no answer.

**Figure 10:**
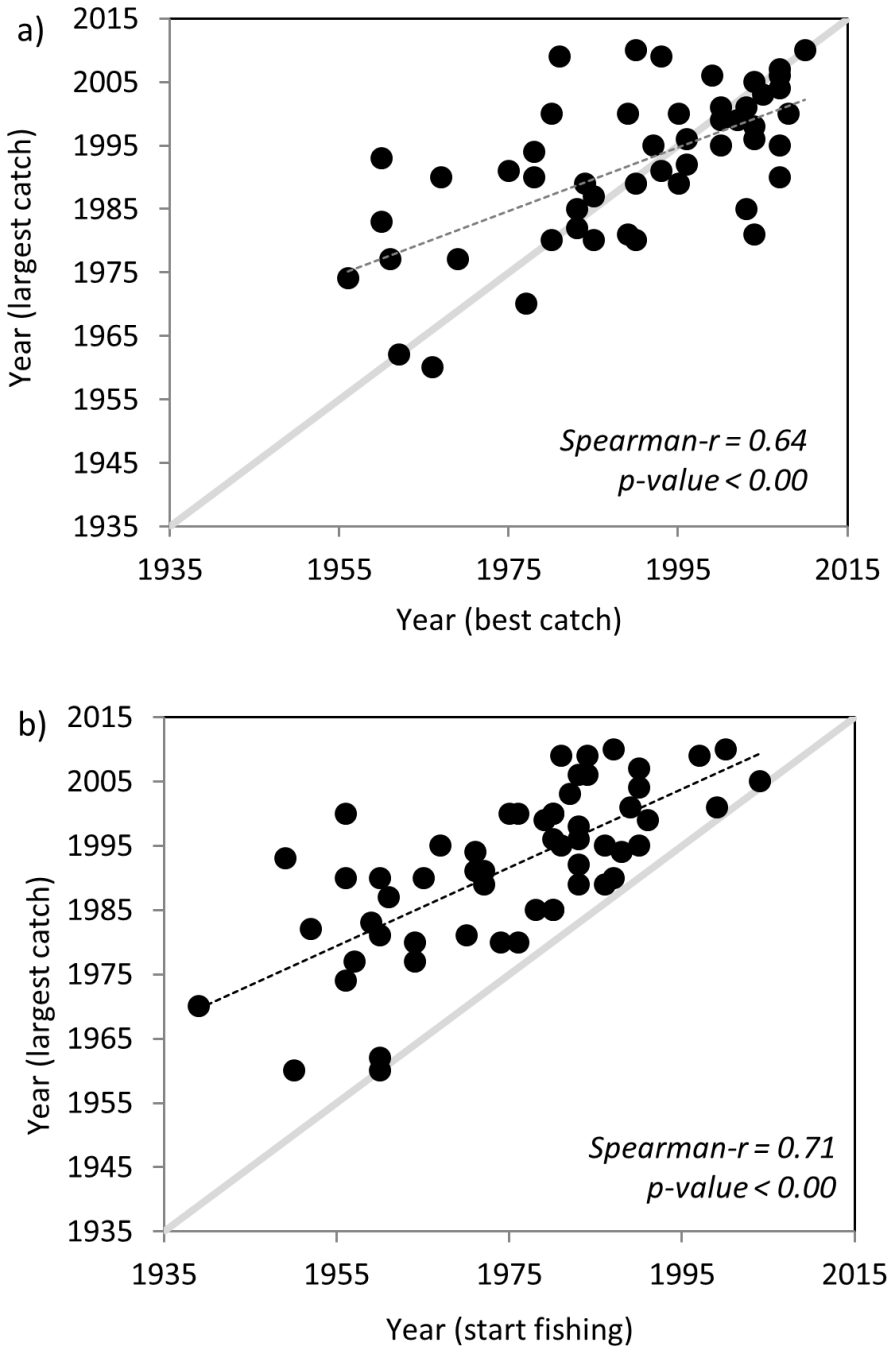
Assessment of the a) year of the best catch and of the largest caught species, and b) year at starting fishing and the largest caught species. Diagonal grey line represents the x = y function.

When comparing the year of the largest catch (best catch) with the year of the largest caught species (largest catch) reported by fishers, a positive and significant relationship (Spearman-r  = 0.64, p-value <0.00) highlighted that years when fishers captured the largest catch tended to coincide with years of the best catch (Figure 10a). However, when the best catch was made before the 1990s, the largest catch was later in fishers' career, while after the 1990s fishers first recorded a largest catch and then a best catch.

When comparing the years of start fishing with the year of the largest catch a positive and also significant relationship (Spearman-r  = 0.71, p-value <0.00) was observed (Figure 10b). This relationship approached the relationship that one would expect under the hypothesis that there is a shifting in the baseline: the year that fishers start fishing is the best year, and afterwards the largest catch declines (a similar and also significant relationship was observed between years of start fishing and best catch, not shown here). These patterns may highlight an expansion of the fishery, followed by depletion and a shift in the ecological baseline of fishers.

### Ecosystem changes

- **Depleted species:** Ninety-four percent of the fishers listed one or more species or groups of species as depleted (Table 1). In total, 76 taxa were listed (Table S2). Of those most frequently listed, the most common were commercial species such as common hake, sardine, anchovy and red mullets (Table 2). Other commercial species followed, such as mackerel, anglerfish, common seabream *Pagrus pagrus* and flatfish *Solea solea*. Fishers also listed invertebrate species, such as the spinous spider crab *Maja squinado* and the spiny lobster, and various species of sharks such as the nursehound *Scyliorhinus stellaris,* the smooth-hound *Mustelus mustelus* and the longnose spurdog *Squalus blainville* (Table 2).

**Table 2:** Summary of taxa and groups listed by fishers as being depleted (D), locally extinct (E), and as having proliferated (P).

Species	Depletion	Extinction	Proliferation
*Aristeus antennatus*	1		1
*Auxis rochei*	1		1
*Belone belone*	2		
*Coryphaena hippurus*	1	2	1
*Dasyatis pastinaca*			2
*Dentex dentex*			2
*Dicentrarchus labrax*	2		
*Engraulis encrasicolus*	10		1
*Epinephelus* spp.	2		
*Helicolenus dactylopterus*	1	1	
*Hippocampus* sp.		3	
*Homarus gammarus*	1	1	
*Lithognathus mormyrus*	2		
*Lophius* spp.	5		1
*Maja squinado*	4	10	
*Merluccius merluccius*	12		1
*Micromesistius poutassou*	3		
*Mullus* spp.	8		6
*Mustelus asterias*		3	
*Mustelus mustelus*	3	2	1
*Nephrops norvegicus*	2		1
*Oxynotus centrina*	2	1	
*Pagellus bogaraveo*	1	3	
*Pagellus erythrinus*	2	1	
*Pagrus auriga*	2		
*Pagrus pagrus*	5		
*Palinurus elephas*	4		
*Plectorhinchus mediterraneus*	2	1	
*Pomadasys incisus*	2		
*Prionace glauca*	1	1	
*Psetta maxima*	2		
Rajiformes	1		2
*Rhinobatos rhinobatos*		6	
*Sarda sarda*	2		
*Sardina pilchardus*	11	1	3
*Sardinella aurita*			3
*Sciaena umbra*	2	3	1
*Scomber* spp.	6		7
*Scorpaena scrofa*	2		
*Scyliorhinus canicula*			2
*Scyliorhinus stellaris*	5	8	
*Scyllarides latus*	1		1
*Seriola dumerili*	3		
*Solea solea*	5		
*Solea* sp.	2		
*Sparisoma cretense*	2		
*Sparus aurata*	2		1
*Spicara* spp.	2		
*Sprattus sprattus*		2	
*Squalus acanthias*	2	1	
*Squalus blainvillei*	3		
*Squatina oculata*	2	4	
*Squatina squatina*		9	
*Torpedo* spp.		2	
*Trachurus* spp.	1		2
*Trichiurus lepturus*	1	1	
*Trisopterus minutus*	2		
*Umbrina cirrosa*		2	
*Zeus faber*	2		2

Groups	Depletion	Extinction	Proliferation
ALL	11		
Cephalopods	1		
Commercial invertebrates	1	1	
Cuttlefish	1		2
Jellyfish			2
Octopus	1		5
Marine turtles	2	1	
Commercial shrimps	3		2
Squids	1	1	2
Whales	1	1	

This table lists species that were mentioned at least twice in a group (D, E or P), or that were listed in two or more groups (Table S2 contains the full list of species).

PERMANOVA analysis highlighted significant differences by region of species identified as depleted (Table 3). SIMPER results showed that while the category “all”, “shrimps”, sardine, common seabream, and blue whiting were the main species identified as depleted in the Gulf of Cadiz, common hake and anchovy were important in the Mediterranean Andalusian side and in the Tramontana region (Table 4). The Balearic Islands showed different species such as two species of sharks (smooth-hound and longnose spurdog), garfish *Belone belone*, and spiny lobster as most cited by fishers to be depleted.

**Table 3:** PERMANOVA analysis by region. D: depletion, E: local extinction, P: proliferation.

Analysis	df	SS	MS	Pseudo-F	P (perm)
**D - region**	3	19375	6458.2	1.49	**0.02**
**E - region**	2	37345	18672	6.20	**0.00**
**P - region**	3	26987	8995.5	2.36	**0.00**

**Table 4:** SIMPER analysis by region and species or groups (Table 2) by depletion, local extinction, and proliferation lists.

Regions	Species or groups	Av. contribution (%)	Cum. contribution (%)
**A. Depletion**			
Gulf of Cadiz	“ALL”	34.7	34.7
	*Sardina pilchardus*	15.2	49.8
	*Pagrus pagrus*	9.8	59.6
	“Shrimps”	9.6	69.2
	*Micromesistius poutassou*	7.7	76.9
Andalusia-Med	*Merluccius merluccius*	54.8	54.8
	*Engraulis encrasicolus*	23.7	78.5
Tramontana	*Sardina pilchardus*	22.1	22.1
	*Merluccius merluccius*	19.7	41.8
	“ALL”	19.3	61.0
	*Engraulis encrasicolus*	11.8	72.8
	*Mullus* spp.	9.9	82.7
Balearic Islands	*Mustelus mustelus*	25.0	25.0
	*Squalus blainvillei*	18.0	42.9
	“ALL”	12.8	55.7
	*Belone belone*	7.7	63.4
	*Mullus* spp.	7.7	71.1
	*Palinurus elephas*	7.7	78.7
**B. Extinctions**			
Gulf of Cadiz	*Rhinobatos rhinobatos*	61.3	61.3
	*Squatina squatina*	17.3	78.6
	*Mustelus asterias*	7.1	85.7
Tramontana	*Sciaena umbra*	67.4	67.4
	*Coryphaena hippurus*	15.0	82.4
	*Sprattus sprattus*	11.2	93.6
Balearic Islands	*Maja squinado*	49.7	49.7
	*Scyliorhinus stellaris*	32.1	81.8
	*Squatina squatina*	17.2	99.0
**C. Proliferations**			
Gulf of Cadiz	*Scomber* spp.	50.0	50.0
	*Mullus* spp.	37.5	87.5
Andalusia-Med	*Scomber* spp.	84.0	84.0
	*Sardinella aurita*	16.0	100.0
Tramontana	“Octopus”	87.5	87.5
	“Cuttlefish”	6.8	94.3
Balearic Islands	*Zeus faber*	55.6	55.6
	Rajiformes	44.4	100.0

- **Local extinctions**: Sixty-one percent of the fishers listed one or more species or groups of species that had locally disappeared (Table 1). In total, 33 taxa were listed (Table S2). Of those most frequently listed, one was an invertebrate, the spinous spider crab, five were sharks (the angelsharks *Squatina squatina* and *S. oculata*, the common guitarfish *Rhinobatos rhinobatos*, the nursehound and the smooth-hound *Mustelus asterias*), and three were fish species (seahorses *Hippocampus* sp., the blackspot seabream *Pagellus bogaraveo*, and the brown meager *Sciaena umbra*). Marine mammals and turtles were listed as generic groups (Table 2).

PERMANOVA analysis highlighted significant differences by region of species identified as locally extinct (Table 3). While in the Gulf of Cadiz and the Balearic Islands main species listed were cartilaginous fish (common guitarfish, angelsharks, smooth-hound and nursehound), in the Tramontana region main species listed were bony fish such as brown meagre, common dolphinfish *Coryphaena hippurus* and European sprat *Sprattus sprattus* (Table 4). Fishers from the Balearic Islands mainly listed the spinous spider crab as locally extinct.

- **Proliferations:** Fifty-three percent of fishers listed species or groups of species that had proliferated (Table 1). In total, 37 taxa were listed (Table S2). Of those species most frequently listed, three highly commercial fish were listed the most: mackerels, red mullets and sardines (Table 2). In addition, round sardinella and “octopuses” were listed several times. Other fish species were listed, such as horse mackerels and John Dory *Zeus faber,* five groups of invertebrates, such as “jellyfish”, “shrimps” and “squids”, and three small-size shark species, such as the small-spotted catshark *Scyliorhinus canicula* and the common stingray *Dasyatis pastinaca*.

PERMANOVA analysis highlighted significant differences by region of species identified as proliferating by fishermen (Table 3). In the Gulf of Cadiz and the Andalusian Mediterranean site, mackerel species were the ones that contributed the most to the differences between regions (Table 4). The rest of species with a high contribution were different in each region. In the Tramontana region two groups of invertebrates were especially relevant (“octopus” and “cuttlefish”).

## Discussion

Our study evidences that fishers perceptions from the Spanish Mediterranean Sea and Gulf of Cadiz contain very useful information about fishing activity and ecosystem changes in the area, in agreement with previous studies conducted in different locations of the Mediterranean Sea [32], [33].

**Figure 11:**
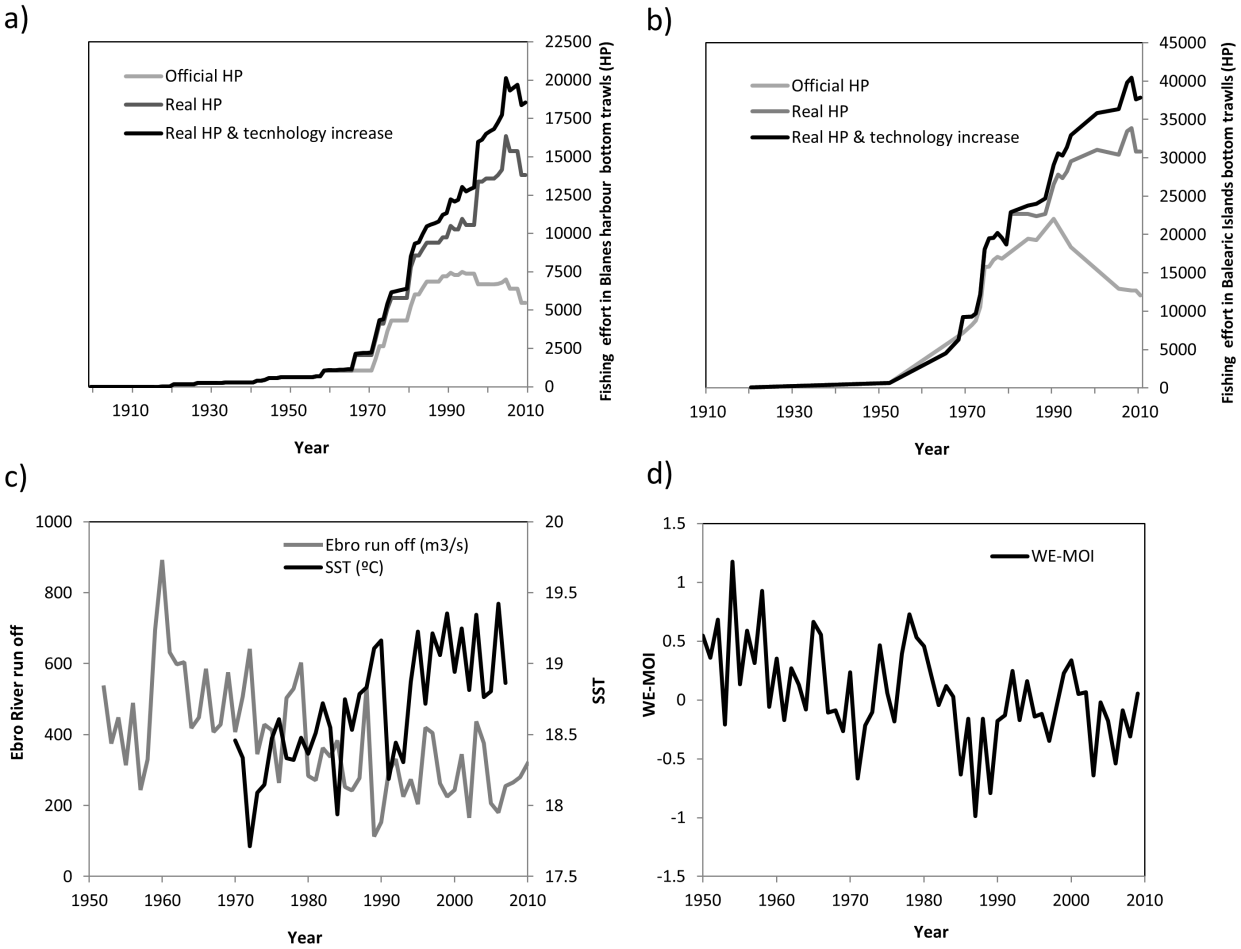
Temporal evolution of a) Bottom trawling fishing effort in the Blanes harbour (NW Mediterranean Sea) from 1900 to 2013 (HP; data sources: Annex S1), b) Bottom trawling fishing effort in the Balearic Islands (NW Mediterranean Sea) from 1920 to 2010 (HP; data sources: Annex S2), c) Ebro River water runoff from 1953 to 2010 (m3/s, annual mean; data sources: Ebro Hydrographical Confederation, Tortosa station,http://www.chebro.es/) and Sea Surface Temperature (SST) from 1970 to 2007 (°C, annual mean; data sources: data was collected near Tarragona, 40°N–2°E, Smith and Reynolds, 2004); and d) Western Mediterranean Oscillation Index from 1950 to 2009 (WEMOI; Error! Hyperlink reference not valid., [76]).

Regarding fishing activities, our results show that fishing in the study area has gone deeper and farther from shore, in line with a general expansion of fishing activities documented worldwide [2], [48], [49] and in the study area [50]–[52]. This was more evident when considering the answers from the elder fishers, suggesting that the most important changes in the ecosystem occurred a few decades ago. Fishers were also able to identify areas where depletion had happened in the study areas, which coincides with other studies documenting changes in marine ecosystems of the Mediterranean Sea [12] and is consistent with the exponential fishing effort growth observed in the study area [50,51,52, and see Figure 11a-b and Annex S1 and S2]. Both results suggesting depletion of resources and the expansion of fishing activities toward deeper and farther waters highlight a progressive depletion of coastal areas in the region.

Fishers reported that the species abundance, measured as the best catch ever and the largest caught species, and the species and size composition of some species in the catch, have changed significantly with time (Figures 6, 7, 8, 9 and Tables 1 and 2). This is in agreement with other studies that use quantitative or qualitative fisheries information in specific areas from the western, central and eastern Mediterranean Sea [e.g., 14,25,53,54]. In addition, and despite our results regarding common hake are not so clear since the weight of the largest common hake ever caught (kg) plotted against the year of starting fishing showed no clear trend, there are evidences that hake has been increasingly subjected to overfishing since the 1980s and nowadays the population is mainly supported by young age classes [55], [56]. In fact, in 2011 the International Union for Conservation of Nature (IUCN) listed the common hake as a vulnerable species in the Mediterranean fish assessment [16] and, hake is assessed as being overexploited in several areas of the Mediterranean [15], [44], [57], [58].

Our results also provide valuable information regarding changes at the species composition level of the exploited ecosystem. Fishers listed several commercial and non-commercial species as depleted. In several cases, these results are in line with various quantitative information available (Table S2) and other regional lists and bibliographic references [59], [60]. Of the 43 species of fish listed as depleted, ten species were listed as data deficient (DD), near threatened (NT), vulnerable (VU) or endangered (EN) by IUCN [16] (Table S2). This represents the 24% of the species listed by fishers. Three of those species were also listed in the Bern and Barcelona conventions [40], [41], and one was assessed as overexploited by the General Fisheries Commission of the Mediterranean Sea (GFCM) [44] (Table S2). Of the shark species listed as depleted, nine of the ten species listed had been listed as DD, NT, VU, EN or critically endangered (CR) by IUCN [16] (Table S2). This represents the 90% of the species listed by fishers and is consistent with the fact that sharks are highly vulnerable to fishing activity both globally [61], [62] and in the Mediterranean Sea [63], [64], and that fishing effort in the study area has grown exponentially (Figure 11a–b, and Annex S1 and S2). Of the invertebrate species listed, three species had been included in the Bern and Barcelona convention [40], [41] as species with exploitation being regulated, and three additional ones were assessed as overexploited by the GFCM [44] (Table S2). Most of these species listed as being depleted by fishers are known to have been highly impacted by fishing [15], [16]. In some cases though, some species may have declined due to additional effects of climate change, as it is the case of sardine and European sprat [65] and the increase sea surface temperature trend in the area (Figure 11c). Changes in the environmental conditions in the study area with time include an increase of sea surface temperature and declines in river runoffs (as exemplified in Figure 11c for the Ebro River) and, in general, but warmer and drier conditions (Figure 11d), with clear effects on marine ecosystems in the region [66].

Regarding locally extinct or extirpated species listed by fishers, the spinous spider crab and the European lobster *Homarus gammarus*, both highly commercial species, were included in the Bern and Barcelona conventions [40], [41]. Of the finfish species listed, three were also listed by IUCN as NT and VU: this is the case of the brown meager, the shi drum *Umbrina cirrosa*, and seahorses [16]. Of shark species, nine of the ten species of sharks listed by fishers were in the IUCN list and three of those in other international agreements [16]. When looking at all fish species listed, 12 of the 21 fish species were IUCN listed [16], and of those six were also listed in other international agreements (so 50% of fish) and one more (sardine) was included in the GFCM assessment as fully exploited or overexploited [44] (Table S2). Locally extinctions or extirpations can mainly be related with an increase of fishing intensity in the area to target commercial species (Figure 11a–b and Annex S1 and Annex S2) and the high vulnerability of some target and non-target species to fishing [15], [16].

It is interesting to note that fishers listed a larger group of species being depleted or locally extinct than official conventions and assessments (Table S2). This may be due to fishers are reporting local depletions and extirpations and thus these results are relevant at a small regional scale. It could also be related to the fact that fishers are the immediate observers of the sea, thus they may list depletions that have not been yet observed by the scientists or are not reported by official documents yet. On the other hand, stock assessment datasets in the Mediterranean Sea are still limited to few highly commercial species (such as common hake, anchovy, sardine, red mullets, Norway lobster and red shrimp) [15], [44] and thus assessments are still missing for many exploited species. It is also recognised that commercial fishes, and also invertebrate species, are more difficult to list in official conventions due to unavailability of data but also economic interests to keep exploiting the species [67].

Fishers also listed species that became more abundant with time, in agreement with independent information from the area [52]. This may be due to these species had benefited from a reduction of predators in some areas (due to predation release or competition decrease), as can be the case of cephalopods or small-sized fish [53], [68]. Other species may have also benefited from changes in physical and oceanographic conditions related to climate change, as it is the case of the round sardinella [69], cephalopods [68] and jellyfish [70] (Figure 11c–d). However, the way that multiple impacts (such as fishing and climate change) interact and combine to impact species and productivity patterns of marine ecosystems is still hardly known [71] and difficult the interpretation of some biodiversity changes. In addition, some benthopelagic and pelagic species listed as proliferating, such as John Dory, could be perceived as having increased due to modifications of the fishing technique (for example, by increasing the height of the bottom trawling net). This issue needs further investigation.

Our results showed some specificity by region as well. This can be due to our study area presents a north to south gradient in the Mediterranean Sea (Figure 1), which is characterized by some differential oceanographic and productivity conditions that can affect the presence and production of species [72]. In addition, fishing effort differences between the Spanish peninsula and the Balearic Islands may explain the fact that more species of cartilaginous fish are still listed in the islands since fishing effort is higher in the mainland [39], [73], [74] (Figure 11a–b and Annex S1 and Annex S2). It is therefore important to highlight that regional dynamics are essential when analysing and interpreting biodiversity changes.

Overall, this study illustrates that traditional knowledge that can be retrieved from local fishers is useful to complement the information available regarding the evolution of fishing activities and changes in marine resources and biodiversity in the Mediterranean Sea. This information can complement the quantitative knowledge generated by fisheries assessments and can be useful to complement quantitative monitoring and evaluation surveys to keep track of fishing and climate change effects in the Mediterranean Sea. This knowledge is essential to set current data from the study area into a historical perspective, and prevent the shifting baseline syndrome [21]–[23]. Such recovery of information is also especially relevant in the context of the new Common Fisheries Policy (CFP) of the European Commission and the Marine Strategy Framework Directive (MSFD), which aims at achieving a Good Environmental Status (GES) in European marine waters by 2020, at the latest [75].

## Supporting Information

Table S1 **Complete list of targeted species listed by fishers.**
(DOCX)

Table S2 **Complete list of taxa listed by fishers as being depleted (D), locally extinct (E), and as having proliferated (P).** The classification of species in different international agreements or stock assessments is indicated.
(DOCX)

Annex S1 **Bottom trawling fishing effort reconstruction of the Blanes fishing harbour, Catalan Sea.**
(DOCX)

Annex S2 **Bottom trawling fishing effort reconstruction of the Balearic Islands.**
(DOCX)
