# Supplementary material for: Assessing Fishing and Marine Biodiversity Changes Using Fishers' Perceptions: The Spanish Mediterranean and Gulf of Cadiz Case Study
Source: PLoS One. 2014 Jan 22;9(1):e85670. doi: 10.1371/journal.pone.0085670 (PMC3899065; doi:10.1371/journal.pone.0085670)
Supplement: Table S1 — Complete list of targeted species listed by fishers. (DOCX) [file pone.0085670.s001.docx]

Supporting Online Information

**Table S1.** Complete list of targeted species listed by fishers.

|  |  |
| --- | --- |
| **Targeted species** | **Listed by fishers (%)** |
| *Mullus* spp. | 51.6% |
| *Merluccius merluccius* | 45.3% |
| *Aristeus antennatus* | 42.2% |
| *Lophius* spp. | 42.2% |
| *Engraulis encrasicolus* | 31.3% |
| *Sardina pilchardus* | 29.7% |
| *Palinurus elephas* | 17.2% |
| *Nephrops norvegicus* | 10.9% |
| *Coryphaena hippurus* | 9.4% |
| *Pagrus pagrus* | 7.8% |
| *Scorpaena scrofa* | 7.8% |
| *Micromesistius poutassou* | 7.8% |
| *Scomber* spp. | 6.3% |
| *Trachurus* spp. | 6.3% |
| *Pagellus* spp. | 6.3% |
| *Solea solea* | 6.3% |
| *Dentex dentex* | 6.3% |
| *Seriola dumerili* | 6.3% |
| *Auxis rochei* | 4.7% |
| *Sparus aurata* | 3.1% |
| *Donax trunculus* | 3.1% |
| *Epinephelus caninus* | 3.1% |
| *Aphya minuta* | 3.1% |
| *Physis* sp. | 3.1% |
| *Zeus faber* | 3.1% |
| *Sarda sarda* | 3.1% |
| *Callista chione* | 1.6% |
| *Penaeus kerathurus* | 1.6% |
| *Chamelea gallina* | 1.6% |
| *Spondyliosoma cantharus* | 1.6% |
| *Naucrates ductor* | 1.6% |
| *Centracanthus cirrus* | 1.6% |
| *Stichopus regalis* | 1.6% |
| **Targeted groups** | **Listed by fishers (%)** |
| Cuttlefish | 10.9% |
| Octopus | 9.4% |
| Fish in general | 7.8% |
| Squids | 4.7% |
