# Supplementary material for: Assessing Fishing and Marine Biodiversity Changes Using Fishers' Perceptions: The Spanish Mediterranean and Gulf of Cadiz Case Study
Source: PLoS One. 2014 Jan 22;9(1):e85670. doi: 10.1371/journal.pone.0085670 (PMC3899065; doi:10.1371/journal.pone.0085670)
Supplement: Table S2 — Complete list of taxa listed by fishers as being depleted (D), locally extinct (E), and as having proliferated (P). The classification of species in different international agreements or stock assessments is indicated. (DOCX) [file pone.0085670.s002.docx]

Supporting Online Information

**Table S2.** Complete list of taxa listed by fishers as being depleted (D), locally extinct (E), and as having proliferated (P). The classification of species in different international agreements or stock assessments is indicated.

|  |  |  |  |  |  |  |  |  |  |
| --- | --- | --- | --- | --- | --- | --- | --- | --- | --- |
|  | **Fishers' responses** | | | **International conventions and stock assessments** | | | | | |
| **Species** | **Depletion** | **Extinction** | **Proliferation** | **IUCN^1**^** | **BERN^2^** | **BARCOM^3^** | **BONN^4^** | **CITES^5^** | **FAO-GFCM^6^** |
| *Argyrosomus regius* | 1 |  |  | LC |  |  |  |  |  |
| *Anguilla anguilla* |  |  |  | CR |  |  |  |  |  |
| *Aristeus antennatus* | 1 |  | 1 |  |  |  |  |  | Over-exploited |
| *Aspitrigla cuculus* |  | 1 |  | LC |  |  |  |  |  |
| *Atherina boyeri* | 1 |  |  | LC |  |  |  |  |  |
| *Auxis rochei* | 1 |  | 1 | LC |  |  |  |  |  |
| *Balistes* sp. |  |  |  | DD |  |  |  |  |  |
| *Belone belone* | 2 |  |  | LC |  |  |  |  |  |
| *Bolinus brandaris* | 1 |  |  |  |  |  |  |  |  |
| *Boops boops* |  |  | 1 | LC |  |  |  |  | Over-exploited |
| *Brama brama* | 1 |  |  | DD |  |  |  |  |  |
| *Buglossidium luteum* | 1 |  | 1 | LC |  |  |  |  |  |
| *Callista chione* | 1 |  |  |  |  |  |  |  |  |
| *Cepola macrophthalma* | 1 |  |  | LC |  |  |  |  |  |
| *Chimaera monstrosa* | 1 |  |  | DD |  |  |  |  |  |
| *Coryphaena hippurus* | 1 | 2 | 1 | LC |  |  |  |  |  |
| *Dasyatis pastinaca* |  |  | 2 | NT |  |  |  |  |  |
| *Dentex dentex* |  |  | 2 | VU |  |  |  |  |  |
| *Dicentrarchus labrax* | 2 |  |  | NT |  |  |  |  |  |
| *Diplodus* spp. |  | 1 |  | LC/DD |  |  |  |  |  |
| *Dipturus oxyrinchus* |  |  | 1 | NT |  |  |  |  |  |
| *Donax trunculus* | 1 |  |  |  |  |  |  |  |  |
| *Eledone cirrhosa* |  |  | 1 |  |  |  |  |  |  |
| *Engraulis encrasicolus* | 10 |  | 1 | LC |  |  |  |  | Moderately  exploited (Alboran) / Fully exploited (North Spain) |
| *Epinephelus marginatus* |  |  | 1 | EN | Appendix III | Annex III |  |  |  |
| *Epinephelus* spp. | 2 |  |  | DD/EN |  |  |  |  |  |
| *Gadus morhua* |  |  |  | VU* |  |  |  |  |  |
| *Helicolenus* dactylopterus | 1 | 1 |  | LC |  |  |  |  |  |
| *Hippocampus* spp. |  | 3 |  | NT | Appendix II |  |  | Appendix II |  |
| *Homarus gammarus* | 1 | 1 |  |  | Appendix III | Annex II |  |  |  |
| *Lampris guttatus* |  |  |  | DD |  |  |  |  |  |
| *Lepidopus caudatus* |  | 1 |  | LC |  |  |  |  |  |
| *Lithognathus mormyrus* | 2 |  |  | LC |  |  |  |  |  |
| *Lophius* spp. | 5 |  | 1 | LC |  |  |  |  |  |
| *Maja squinado* | 4 | 10 |  |  | Appendix III | Annex III |  |  |  |
| *Merluccius merluccius* | 12 |  | 1 | VU |  |  |  |  | Over-exploited |
| *Micromesistius poutassou* | 3 |  |  | LC |  |  |  |  |  |
| *Mullus* spp. | 8 |  | 6 | LC |  |  |  |  | Over-exploited |
| *Munida* spp. | 1 |  |  |  |  |  |  |  |  |
| *Mustelus asterias* |  | 3 |  | EN |  |  |  |  |  |
| *Mustelus mustelus* | 3 | 2 | 1 | EN |  |  |  |  |  |
| *Nephrops norvegicus* | 2 |  | 1 |  |  |  |  |  | Over-exploited |
| *Oxynotus centrina* | 2 | 1 |  | CR |  |  |  |  |  |
| *Pagellus acarne* |  |  | 1 | LC |  |  |  |  |  |
| *Pagellus bogaraveo* | 1 | 3 |  | LC |  |  |  |  | Over-exploited |
| *Pagellus erythrinus* | 2 | 1 |  | LC |  |  |  |  | Fully exploited |
| *Pagellus* sp. |  | 1 |  | LC |  |  |  |  |  |
| *Pagrus auriga* | 2 |  |  | DD |  |  |  |  |  |
| *Pagrus pagrus* | 5 |  |  | LC |  |  |  |  |  |
| *Palinurus elephas* | 4 |  |  |  | Appendix III | Annex III |  |  |  |
| *Parapenaeus longirostris* | 1 |  |  |  |  |  |  |  | Over-exploited |
| *Penaeus kerathurus* |  |  | 1 |  |  |  |  |  |  |
| *Phycis* sp. | 1 |  |  | LC |  |  |  |  |  |
| *Plectorhinchus mediterraneus* | 2 | 1 |  | LC |  |  |  |  |  |
| *Plesiopenaeus edwardsianus* |  | 1 |  |  |  |  |  |  |  |
| *Pomadasys incisus* | 2 |  |  | LC |  |  |  |  |  |
| *Pomatomus saltatrix* | 1 |  |  | LC |  |  |  |  |  |
| *Prionace glauca* | 1 | 1 |  | VU | Appendix III | Annex III |  |  |  |
| *Psetta maxima* | 2 |  |  | NT |  |  |  |  |  |
| *Raja clavata* | 1 |  |  | NT |  |  |  |  |  |
| *Raja* spp. | 1 |  | 2 | CR/EN/NT/LC/DD | |  |  |  |  |
| *Rhinobatos rhinobatos* |  | 6 |  | EN, B1 |  |  |  |  |  |
| *Rhizostoma* sp. |  |  |  |  |  |  |  |  |  |
| *Sarda sarda* | 2 |  |  | LC |  |  |  |  |  |
| *Sardina pilchardus* | 11 | 1 | 3 | LC |  |  |  |  | Fully exploited (Alboran) / Overexploited (North Spain) |
| *Sardinella aurita* |  |  | 3 | LC |  |  |  |  |  |
| *Sciaena umbra* | 2 | 3 | 1 | VU | Appendix III | Annex III |  |  |  |
| *Scomber* spp. | 6 |  | 7 | NT/LC |  |  |  |  |  |
| *Scophthalmus rhombus* |  |  |  | LC |  |  |  |  |  |
| *Scorpaena scrofa* | 2 |  |  | LC |  |  |  |  |  |
| *Scyliorhinus canicula* |  |  | 2 | LC |  |  |  |  |  |
| *Scyliorhinus stellaris* | 5 | 8 |  | NT, B2 |  |  |  |  |  |
| *Scyllarides latus* | 1 |  | 1 |  |  |  |  |  |  |
| *Seriola dumerili* | 3 |  |  | LC |  |  |  |  |  |
| *Solea solea* | 5 |  |  | LC |  |  |  |  |  |
| *Solea* sp. | 2 |  |  | LC |  |  |  |  |  |
| *Sparisoma cretense* | 2 |  |  | LC |  |  |  |  |  |
| *Sparus aurata* | 2 |  | 1 | LC |  |  |  |  |  |
| *Spicara* spp. | 2 |  |  | LC |  |  |  |  |  |
| *Sprattus sprattus* |  | 2 |  | DD |  |  |  |  |  |
| *Squalus acanthias* | 2 | 1 |  | EN, B3 |  | Annex II | Appendix II |  |  |
| *Squalus blainvillei* | 3 |  |  | DD, B4 |  |  |  |  |  |
| *Squatina oculata* | 2 | 4 |  | CR |  |  |  |  |  |
| *Squatina squatina* |  | 9 |  | CR | Appendix III | Annex III |  |  |  |
| *Squilla mantis* | 1 |  |  |  |  |  |  |  |  |
| *Torpedo* spp. |  | 2 |  | DD/LC |  |  |  |  |  |
| *Trachurus* spp. | 1 |  | 2 | LC |  |  |  |  | Fully exploited |
| *Trichiurus lepturus* | 1 | 1 |  | LC |  |  |  |  |  |
| *Chelidonichthys lucernus* |  | 1 |  | LC |  |  |  |  |  |
| Triglidae |  |  | 1 | LC |  |  |  |  |  |
| *Trisopterus minutus* | 2 |  |  | LC |  |  |  |  |  |
| *Umbrina cirrosa* |  | 2 |  | VU | Appendix III | Annex III |  |  |  |
| *Xiphias gladius* | 1 |  |  | NT |  | Annex III |  |  |  |
| *Thunnus thynnus* | 1 |  |  | EN |  | Annex III |  |  |  |
| *Zeus faber* | 2 |  | 2 | LC |  |  |  |  |  |
| **Groups** | **Depletion** | **Extinction** | **Proliferation** |  |  |  |  |  |  |
| ALL | 11 |  |  |  |  |  |  |  |  |
| Cephalopods | 1 |  |  |  |  |  |  |  |  |
| Commercial invertebrates | 1 | 1 |  |  |  |  |  |  |  |
| Cuttlefish | 1 |  | 2 |  |  |  |  |  |  |
| Jellyfish |  |  | 2 |  |  |  |  |  |  |
| Marine turtles | 2 | 1 |  |  |  |  |  |  |  |
| Octopus | 1 |  | 5 |  |  |  |  |  |  |
| Shrimps | 3 |  | 2 |  |  |  |  |  |  |
| Small pelagic fish | 1 |  |  |  |  |  |  |  |  |
| Small tuna |  |  | 1 |  |  |  |  |  |  |
| Squids | 1 | 1 | 2 |  |  |  |  |  |  |
| Whales | 1 | 1 |  |  |  |  |  |  |  |
| *Not a Mediterranean species, ** The IUCN Red List of Threatened Species™ – Regional Assessment for the Mediterranean Sea (Abdul Malak et al. 2011). | | | | | | | | | |
| DD = Data Deficient, LC = Least Concern, VU = Vulnerable, NT = Near Threatened, EN = Endangered, CR = Critically Endangered.  B1: Classified as extinct in the Balearic Islands, B2: Classified as EN in the Balearic Islands, B3: Classified as CR in the Balearic Islands, B4: Classified as VU in the Balearic Islands (Source: Mayol, J., Grau, A., Riera, F., Oliver, J., 2000. Llista vermella dels peixos de les Balears. Conselleria de Medi Ambient i Conselleria d’Agricultura i Pesca, Mallorca 127 pp.).  1 International Union for Conservation of Nature and Natural Resources, 2 Bern Convention: Convention on the Conservation of European Wildlife and Natural Habitats (1979), 3 Barcelona Convention: Convention for the Protection of the Marine Environment and the Coastal Region of the Mediterranean (1976, amended in 1995), Protocol Concerning specially Protected Areas and Biological Diversity in the Mediterranean (SAP-Bio) (1995), 4 Bonn Convention: Convention on the Conservation of Migratory Species of Wild Animals (1983), 5 CITES: Convention on International Trade in Endangered Species of Wild Fauna and Flora (1975), 6 General Fisheries Commission for the Mediterranean Sea - Scientific Advisory Committee GFCM:SAC13/2011/3. | | | | | | | | | |
|  | | | | | | | | | |
|  |  |  |  |  |  |  |  |  |  |
|  |  |  |  |  |  |  |  |  |  |
