# Supplementary material for: Assessing Fishing and Marine Biodiversity Changes Using Fishers' Perceptions: The Spanish Mediterranean and Gulf of Cadiz Case Study
Source: PLoS One. 2014 Jan 22;9(1):e85670. doi: 10.1371/journal.pone.0085670 (PMC3899065; doi:10.1371/journal.pone.0085670)
Supplement: Annex S1 — Bottom trawling fishing effort reconstruction of the Blanes fishing harbour, Catalan Sea. (DOCX) [file pone.0085670.s003.docx]

Supporting Online Information

**Annex 1**. Bottom trawling fishing effort reconstruction of the Blanes fishing harbour, Catalan Sea

Following previous initiatives [[1](#_ENREF_1)] to reconstruct historical series of fishing effort, we obtained official data on the fleets of all the ports of Catalonia from the beginning of the last century to June 2013 from the Spanish Ministry of Agriculture, Livestock and Fishery. These data provided information on the individual vessels on the type of vessel, period of activity, vessel size, and engine horse power (hp). We used fishing effort for the trawling fleet of the port of Blanes using the vessel’s hp values. The first historical series (Figure 11a in the main body of the manuscript, official HP series) was built summing up the hp official values of all trawlers by year. The problem that we faced working with official data is that the hp values were often under reported, especially when in reality they exceeded the 500 hp limit set by law. Also, changes to increase the engine power after the construction of vessels were not reported in the official database, so the evolution of fishing effort was impossible to be fully outlined.

Therefore, we complemented the official data by performing personal interviews to members of the local Fishermen Association in Blanes harbor (Giulia Gorelli unpublished data). From the interviews, we obtained values of real engine power of all the vessels composing the trawling fleet of the Blanes harbor in 2012-2013, and the information on engine power improvements performed in the vessels in the past. Using this information we improved the official data and we built the historical series of real fishing effort (Figure 11a in the main body of the manuscript, real HP series).

Finally, a 1% yearly increase due to improved fishing technology (net sensors, scanmar, new bathymetry data…) was applied to the series of real fishing effort starting from 1980 in order to simulate the increase in the efficiency of estimated fishing effort (Figure 11a, Real HP & technology increase series). We applied an annual increase of 1% as a conservative ratio respect to the 1.8% previously suggested [[2](#_ENREF_2)].

**References**

1. McCluskey SM, R.L. L (2008) Quantifying fishing effort: a synthesis of current methods and their applications. Fish and Fisheries 9: 188-200.

2. Pauly D, Palomares MLD (2010) An empirical equation to predict annual increases in fishing efficiency. Fisheries Centre University of British Columbia Working Paper Series 07.
