# Supplementary material for: Assessing Fishing and Marine Biodiversity Changes Using Fishers' Perceptions: The Spanish Mediterranean and Gulf of Cadiz Case Study
Source: PLoS One. 2014 Jan 22;9(1):e85670. doi: 10.1371/journal.pone.0085670 (PMC3899065; doi:10.1371/journal.pone.0085670)
Supplement: Annex S2 — Bottom trawling fishing effort reconstruction of the Balearic Islands. (DOCX) [file pone.0085670.s004.docx]

Supporting Online Information

**Annex 2.** Bottom trawling fishing effort reconstruction of the Balearic Islands

To estimate the evolution of bottom trawl fishing effort in the Balearic Islands from 1920 to 2010, we have gathered information on engine power (hp) from several sources. The beginning of our time series is determined by the start of the use of engines in fishing vessels in the Balearic Islands in 1920 [[1](#_ENREF_1)]. Before 1920, bottom trawlers were composed of pairs of sailboats. We gathered the available data regarding bottom trawl official vessel power from several references for available years [[2-7](#_ENREF_2)] (Figure 11b, Official HP).

However, it is well known that at present bottom trawlers in the Balearic Islands have a much higher fishing effort than officially register [[8](#_ENREF_8)], surpassing the established legal limit of 500 HP^^[[1]](#footnote-1)^^ per vessel. The engine power per vessel can frequently be double the permitted by law [[9-11](#_ENREF_9)], whereas official data is between 150 to 320 HP per vessel, frequently. To estimate the real engine power per vessel, we used the data estimated from 1965 to 2008 [[12](#_ENREF_12)]. From 1920 to 1964, we assumed that the real engine power and the official one was the same because it is from 1988 when the regulation of the engine power limit on 500 hp1 per vessel is set up and we think that there were no reason to hide this information before. Then, we gathered information from several references about the bottom trawling fleet along the studied time series [[3-5](#_ENREF_3),[13-19](#_ENREF_13)], which allowed us to calculate the real engine power of the Balearic bottom trawling fleet (Figure 11b, Real HP).

Finally, a 1% yearly increase due to improved fishing technology (e.g., net sensors, scanmar, new bathymetry data, etc.) was applied to the series of real fishing effort starting from 1980 in order to simulate the increase in the efficiency of estimated fishing effort (Figure 11b, Real HP & technology increase series). We applied an annual increase of 1% as a conservative ratio respect to the 1.8% previously suggested [[20](#_ENREF_20)].

**References**

1. Darder J, Oliver P (2007) Els inicis de la pesca al Bou a Alcúdia. V Jornades d’Estudis Locals d’Alcúdia. Ajuntament d’Alcúdia.

2. BP. (1921) Boletín de Pescas. Ministerio de Marina. Dirección General de Navegación y Pesca. Publicado con el concurso del Instituto Español de Oceanografía. Septiembre, Octubre y Noviembre.

3. Velasco T (1992) La flota pesquera de las Islas Baleares. Revista de Geografía XXVI: 67-86.

4. Oliver P (1983) Los recursos pesqueros del Mediterráneo. Primera Parte: Mediterráneo occidental. FAO, Análisis y estudios, 59, 139 p.

5. Oliver P (1991) Bases técnicas para la regulación de la pesca de arrastre en el Mediterráneo. Palma de Mallorca, 3-5 de junio de 1985. Ministerio de Agricultura Pesca y Alimentación. Madrid.

6. Massutí M (1995) La pesca en el Mar Balear. Edicions Cort. Palma de Mallorca, 460 p.

7. MAGRAMA. (2008-2010) Ministerio de Agricultura, Alimentación y Medio Ambiente: National agency (2008-2010). Spanish fleet census.

8. Morales Nin B (2003) Deep water fisheries of the North western Mediterranean. ACP - EU Fisheries Research Report Number 5. Community Research & Development Information Service (CORDIS). The European Commission Community Research.

9. Guijarro B, Gonzalez N, Massutí E (2010) Stock assessment on Parapenaeus longirostris in Balearic Islands (GSA 05). SAC GFCM Sub Committee on Stock Assessment, 46 p.

10. Guijarro B, Ordines F, Massutí E (2010) Stock assessment on Merluccius merluccius in Balearic Islands (GSA 05). SAC GFCM Sub Committee on Stock Assessment, 46 p.

11. Guijarro B, Valls M, Massutí E (2010) Stock assessment on Nephrops norvegicus in Balearic Islands (GSA 05). SAC GFCM Sub Committee on Stock Assessment, 46 p.

12. Quetglas A, Ordines F, Hidalgo M, Monserrat S, Ruiz S, et al. (2013) Synchronous combined effects of fishing and climate within a demersal community. ICES Journal of Marine Science 70: 319-328.

13. MM. (1921) Dirección General de Navegación y Pesca. Boletín de Pescas. Publicado con el concurso del Instituto Español de Oceanografía. Septiembre, Octubre y Noviembre.

14. DGPM. (1952-1960) Estadística de pesca. Ministerio de Comercio.

15. GFCM. (1970) Living deep water resources of the Western Mediterranean and their explotation. Studies and Reviews 44.

16. Massutí M (1989) El Libro Azul de la Pesca Balear. Conselleria de Agricultura i Pesca, 253 p.

17. Massutí M (1994) Els recursos pesquers del Mar Balear. Bases per a una explotació sostenible. Conselleria d’Agricultura i Pesca. Direcció General de Pesca i Cultius Marins, 172 p.

18. CPAAP. (2007-2010) Conselleria de Presidència, Àrea d’Agricultura i Pesca. Estadístiques bàsiques de l’agricultura, la ramaderia i la pesca a les Illes Balears, 2007-2010.

19. Carreras C, Cardona L, Aguilar A (2004) Incidental catch of the loggerhead turtle Caretta caretta off the Balearic Islands (Western Mediterranean). Biological Conservation 117: 321-329.

20. Pauly D, Palomares MLD (2010) An empirical equation to predict annual increases in fishing efficiency. Fisheries Centre University of British Columbia Working Paper Series 07.

1. Real Decreto 679/1988, de 25 de junio, por el que se regula el ejercicio de la pesca de arrastre de fondo en el Mediterráneo. BOE núm. 160, de 5 de julio. [↑](#footnote-ref-1)
